# Effect of laparoscopic cystectomy on ovarian reserve in patients with ovarian cyst

**DOI:** 10.3389/fendo.2022.964229

**Published:** 2022-08-30

**Authors:** Ghazal Mansouri, Maryam Safinataj, Armita Shahesmaeili, Leila Allahqoli, Hamid Salehiniya, Ibrahim Alkatout

**Affiliations:** ^1^ Department of Obstetrics and Gynecology, School of Medicine, Afzalipour Hospital, Kerman University of Medical Sciences, Kerman, Iran; ^2^ HIV/STI Surveillance Research Center, and World Health Organization (WHO) Collaborating Center for HIV Surveillance, Institute for Futures Studies in Health, Kerman University of Medical Sciences, Kerman, Iran; ^3^ Midwifery Department, Ministry of Health and Medical Education, Tehran, Iran; ^4^ Social Determinants of Health Research Center, Birjand University of Medical Sciences, Birjand, Iran; ^5^ Department of Obstetrics and Gynecology, University Hospital Schleswig-Holstein, Kiel, Germany

**Keywords:** Anti-Mullerian hormone, ovarian cyst, laparoscopy, reserve, laparoscopic cystectomy

## Abstract

**Objective:**

This study aimed to evaluate the effect of laparoscopic cystectomy on ovarian reserve in patients with ovarian cysts.

**Material and methods:**

This prospective study was performed on 113 women with ovarian cysts in a tertiary referral teaching hospital. All patients underwent laparoscopic cystectomy. Serum levels of antimüllerian hormone (AMH) were measured pre-and, 3 months postoperatively. The primary outcome of the study was to assess the effect of laparoscopic cystectomy on ovarian reserve based on alterations in serum AMH levels. The secondary outcome of the present study was to evaluate the impact of the number of cauterizations, size and type of cysts, bilaterality (bilateral or unilateral), age, and body mass index (BMI) on the ovarian reserve after laparoscopic excision.

**Results:**

Laparoscopic cystectomy reduced the serum AMH levels preoperatively (1.32 ± 4.48 ng/ml) to postoperatively (3.2 ± 1.93 ng/ml) and the difference (- 1.28 ng/ml) was statistically different (0.001 >P). There was a negative significant relationship between the number of cauterizations used and postoperative serum AMH levels (p ≤ 0.001). There was a significant relationship between the location (p ≤ 0.01), type of cyst (p ≤ 0.001) and the serum AMH levels reduction.

**Conclusion:**

The number of cauterizations used during surgery, the type of cyst, and bilaterality can affect AMH levels that need to be addressed.

## Introduction

The ovarian cyst is found in about 5-15% of reproductive-aged women (1). Ovarian cysts are fluid-filled ovarian spaces that usually begin during the reproductive years (2, 3), and are usually asymptomatic. If symptomatic they are associated with pelvic pain and pressure, and menstrual irregularities (2). Ovarian cysts can be complicated by rupture, hemorrhage, and torsion, which are considered gynecological emergencies (4). Ovarian cyst treatment varies based on the type of cyst, its size, and imaging findings (from maintenance treatments to surgery) (5). Laparoscopic cystectomy has become the gold standard in the surgical management of persistent adnexal masses due to the risks of ovarian cysts present, potential rupture or malignancy (6, 7). Thus, ovarian cystectomy affects ovarian reserves (8–10). Surgical procedures on the ovaries lead to ovarian tissue damage, which can exacerbate the harm to the remaining follicles, which raises concerns for gynecological researchers regarding the use of different surgical procedures in this field (11, 12). The number of the remaining oocytes is called “ovarian reserve” which indicates the functional potential of the ovary, and the number and quality of ovarian follicles (13). Assessment of the levels of anti-Mullerian hormone (AMH), follicle-stimulating hormone (FSH), estradiol, luteinizing hormone (LH), FSH/LH, and Inhibin-B are ovarian reserve tests (14, 15). AMH is a glycoprotein dimer secreted by granulosa cells of the antral primary follicles. Serum AMH level is a good option for testing ovarian reserve due to minimal fluctuations throughout the monthly menstrual cycle (16). On the other hand, some recent national and international studies outcomes indicated a significant decrease in AMH levels during the third and sixth months after ovarian cystectomy as compared to preoperative time (17–19). The effect of factors such as cyst size, pathology, and type of involvement (unilateral, bilateral) for gynecological researchers is always a challenging issue (20). Moreover, none of the similar studies has so far evaluated the effect of intraoperative cauterization on serum AMH levels. Therefore, this study aimed to evaluate the effect of laparoscopic cystectomy on ovarian reserve in patients with ovarian cysts.

## Material and methods

### Study design and patients

This prospective clinical study was conducted in the Afzalipour tertiary referral teaching hospital affiliated to Kerman University of Medical Sciences, Kerman, Iran, from July 2020 to October 2021. This research was approved by the ethics committee of Kerman University of Medical Sciences with the ethics code IR.KMU.AH.1399.40. The main purpose of the study was to evaluate the effects of laparoscopic cystectomy on ovarian reserve in patients with ovarian cysts. Based on the data reported by Alborzi and co-workers (20), and the mean and standard deviation of serum level of AMH before and post-surgery (μ_1 =_ 3.86 ± 3.58 ng/mL and μ _2 =_ 2.06 ± 2.5 ng/mL), a minimum sample size of 110 was determined, with an alpha error of 0.05 and a study power of 80%. As 10% was added to compensate for the probability of loss to follow-up, the final targeted sample size consisted of nearly 120 patients. In this present study, women with endometriosis who had severe pain, women with dermoid cysts, or women with simple ovarian cysts who lacked a response to pharmacological treatment or had cysts larger than 10 cm were candidates to undergo a cystectomy.

Patients who fulfilled the following inclusion criteria were eligible for the study: 1) age 18 - 45 years; 2) body mass index (BMI) 18.5–29.9 kg/m^2^; 3) women with regular menses; 4) no use of hormonal drugs (such as gonadotropin-releasing hormone analogs or oral contraceptives) in the preceding three months and during the study period, and 5) no history of previous ovarian surgery, chemotherapy or pelvic radiotherapy, 6) no history of polycystic ovary syndrome, 7) no history of endocrinologic diseases such as hyperprolactinemia, hypothyroidism or hyperthyroidism. 8) and no evidence of premature ovarian failure (POF) or premature menopause, 9) no suspected or proven genital or extragenital malignancy

Prior to enrollment, the study objectives and steps were explained to the patients. Those who were willing to participate were asked to sign the written consent form. Patients who fulfilled the inclusion criteria and consented to participate in the study were enrolled by a convenient sampling method.

**Figure 1:**
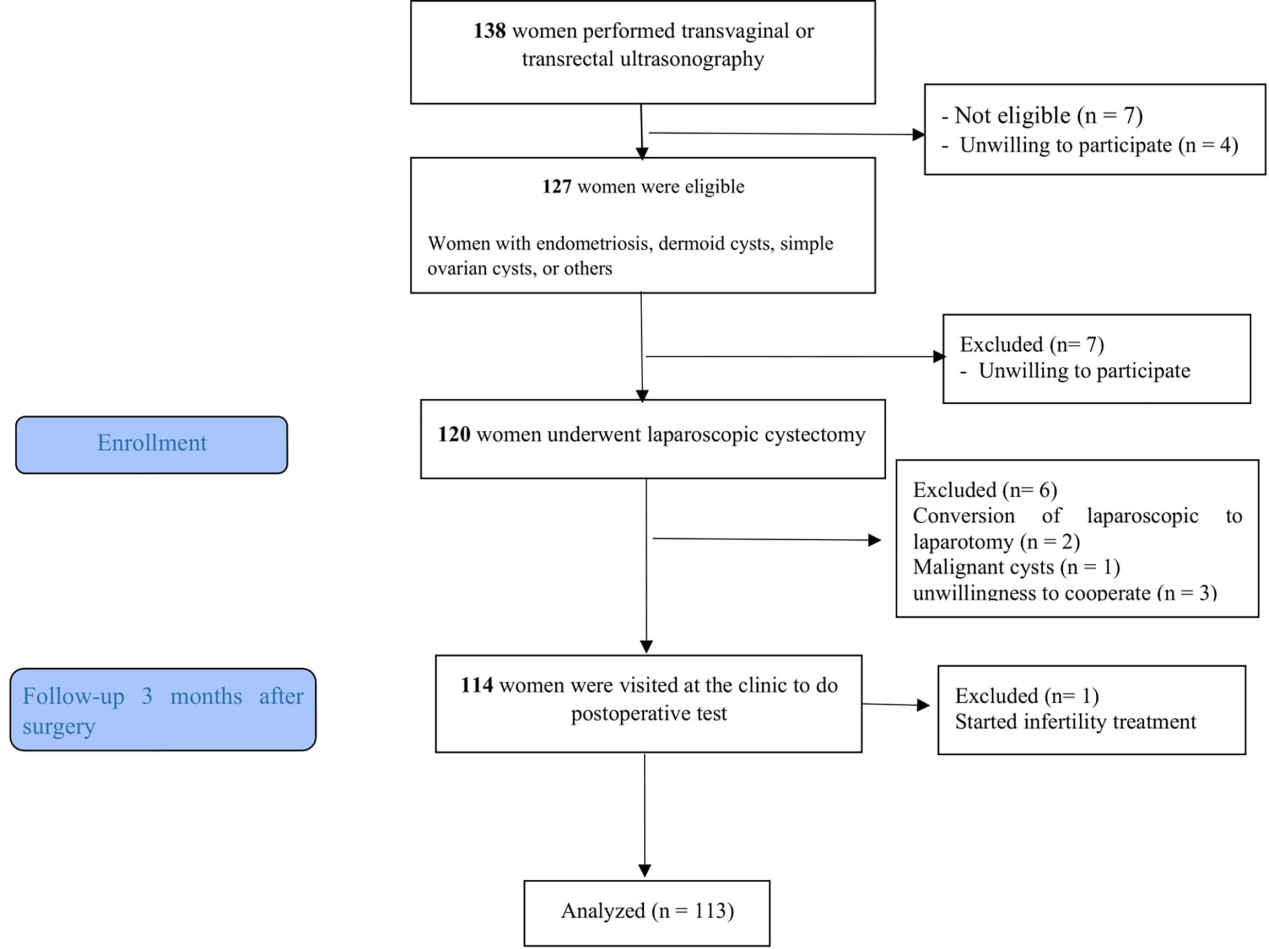
Flowchart of the study.

Women with the conversion from laparoscopic to laparotomy, who had suspected or proven malignant cysts, were unwilling to continue their participation, and started infertility treatment during the study were excluded from the study (**Figure 1**).

### Procedures

Between the third and sixth day of the menstrual cycle, transvaginal or transrectal ultrasonography was performed on all patients to determine the type, and size, and location of the cyst at the same radiology department by a single radiologist. After determining the type, size, and location of the cysts all participants underwent laparoscopic surgery. The day before surgery, 10 ml of cubital vein blood was taken to determine preoperative serum AMH level. The serum level of AMH was measured by the Enzyme-Linked Immunosorbent Assay (ELISA) method, (ELISA kit/AMH MIS HBMG, DRG instrument, Germany) and was reported in ng/ml with a detection limit of 0.006 ng/ml. All hormonal tests were performed in the same reference laboratory using the same assay.

All the patients underwent laparoscopic cystectomy by a laparoscopic team consisting of two experienced gynecologic surgeons under general anesthesia with Storz endoscopic instruments (Karl Storz). The same laparoscopic instruments were used and similar safety principles were followed during surgery. Through a subumbilical incision, pneumoperitoneum was produced by insertion of Verres needle. A 10-mm trocar and telescope were inserted and then three other trocars were introduced. The pelvic cavity was explored. After a sharp incision in the posterior surface of the cyst, ovarian cystectomy was performed by applying traction and contraction forces by two atrophic grasping forceps. Homeostasis was achieved by bipolar coagulation set to 30-40 W *(Karl Storz Company, Clermont-Ferrand* model, Tuttlingen, Germany*)* as each cauterization was applied in about 3-5 seconds. Finally, resected samples were sent to the pathology laboratory for further evaluation and diagnosis. All pathology laboratory testing was performed in the same reference laboratory.

### Outcomes, measurements, and follow up

The primary outcome of the study was to assess the effect of laparoscopic cystectomy on ovarian reserve based on alterations in serum AMH levels. The secondary outcome of the present study was to evaluate the impact of the number of cauterizations, size, location, type of cysts, age, and body mass index (BMI) on the ovarian reserve after laparoscopic excision.

Before the surgery, general and clinical parameters including age, gravidity, parity, weight, height, and BMI were either obtained directly from the patients or extracted from their medical records.

The size of the cysts, bilaterality and the number of cauterizations during the laparoscopic cystectomy were taken into account. Cyst size (> 7cm and ≥7), bilaterality (bilateral or unilateral), and the number of cauterization (≥ 1 time, 2 - 4 times, < 4 times) were recorded during surgery. The type of cyst (endometrioma, dermoid cyst, serous cystadenoma, and others) was determined by pathology laboratory test.

All patients were followed and visited at the clinic three months postoperatively and serum levels of AMH were measured at that time. The preoperative and postoperative serum AMH levels of patients were compared. Also, the differences in serum AMH levels were compared based on the size, and location of the cyst. In addition, the differences between pre-post operative in serum AMH levels were compared based on the number of cauterizations, size, location, type of cysts, age, and BMI.

### Statistical analysis

All data were entered into the statistical software program IBM SPSS Statistics for Windows, version 21.0 (IBM Corp. 2012. Armonk, NY: IBM Corp). Qualitative variables were described by frequency (percentage). Quantitative variables were described using mean and standard deviation (SD). Paired T-test was used to compare serum AMH levels before and after surgery. Student’s t-test and one-way ANOVA tests were used to compare the average decrease in serum AMH level in the studied subgroups. Providing that the ANOVA test was significant, the pairwise comparisons were made with the *Post Hoc* Tukey’s range test. A multiple linear regression model was used to identify predicting factors amount reduction of serum AMH levels. The level of significance was set to 0.05.

## Results

Out of 120 participants, 7 participants were excluded due to, conversion of laparoscopic to laparotomy (n = 2), malignant cysts (n = 1), unwillingness to cooperate in the continuation of the study (not referring to postoperative testing) (n= 3), and started infertility treatment during the study (n = 1) were excluded from the study (**Figure 1**). In total, we included 113 patients. The mean age and BMI of patients were 31.27 ± 7.95 years and 26.68 ± 3.27 kg/m^2^ respectively. The demographic and clinical characteristics of patients are summarized in **Table 1**.

**Table 1:** Demographic and clinical characteristics of 113 women with ovarian cyst undergoing laparoscopic cystectomy.

Variable		
Age, y,	Min - Max	17 - 41
	Mean ± SD	31.27 ± 7.95
BMI, kg.m^2^	Min - Max	17 - 29.8
	Mean ± SD	26.68 ± 3.27
Gravidity, n (%)	≥ 1 2 3 ≤ 4	56 (49.5) 29 (25.6) 15 (13.2) 13 (11.5)
Parity, n (%)	0 1 2 3 ≤ 4	31 (27.4) 26 (23) 31 (27.5) 15 (13.2) 10 (8.8)

Y, Year; SD, Standard deviation; BMI, Body mass index; kg, Kilogram; cm, Centimeter; n, Number.

As a primary outcome of the study, alterations in serum AMH levels were assessed pre-and 3 months postoperatively. The mean serum AMH level decreased before surgery (4.48 ± 1.32 ng/mL) to 3 months (3.2 ± 1.93 ng/mL) postoperatively which was statistically significant (p ≤ 0.001) (**Table 2**).

**Table 2:** Comparison of serum AMH preoperative and 3 months postoperative in different groups.

Variables		n (%)	preoperative	Postoperative 3 months	MD†	P-value††
AMH level, ng/ml		113 (100)	4.48 ± 1.32	3.20 ± 1.93	1.28 ± 0.93	> 0.001^*^
**Number of cauterizations,** n	≥ 1	28 (24.8)	5.05 ± 1.02	4.64 ± 1.22	0.42 ± 0.4	> 0.001^**^
	2 - 4	30 (26.5)	4.73 ± 1.38	3.86 ± 1.58	0.86 ± 0.56	
	< 4	55 (48.7)	4.06 ± 1.31	2.11 ± 1.77	1.95 ± 0.76	
Cyst size, cm	> 7	47 (41.2)	4.40 ± 1.29	3.02 ± 1.95	1.38 ± 1	0.34*^**^
	≤ 7	66 (58.4)	4.5 ± 1.35	3.30 ± 1.93	1.21 ± 0.87	
Bilaterality	Unilateral	105 (92.9)	4.57 ± 1.25	3.35 ± 1.82	1.21 ± 0.92	0.01^***^
	Bilateral	8 (7.1)	3.26 ± 1.67	2.33 ± 1.17	2.08 ± 0.83	
Type of cyst	Serous cystadenoma	9 (8)	4.35 ± 1.97	2.83 ± 1.94	1.51 ± 0.65	>0.001^**^
	Dermoid	29 (25.7)	5.18 ± 1.24	4.38 ± 1.10	0.79 ± 0.68	
	Endometrium	41 (36.3)	4.57 ± 1.25	1.40 ± 1.14	2.09 ± 0.7	
	Other	34 (30.1)	5.11 ± 1.08	4.46 ± 1.42	0.65 ± 0.63	
Age, y	≥ 35	74 (65.4)	4.60 ± 1.32	3.44 ± 1.97	1.24 ± 0.95	0.61^***^
	< 35	39 (34.6)	4.09 ± 1.24	2.75 ± 1.79	1.34 ± 0.95	
BMI, kg.m^2^	≥ 25	58 (51.4)	4.25 ± 1.56	2.90 ± 1.97	1.49 ± 0.54	0.38^***^
	< 25	55 (48.6)	4.49 ± 1.29	3.13 ± 1.54	1.22 ± 0.73	

AMH, Anti-müllerian hormone; MD, Mean difference; ng**/**ml, Nanograms/millilitre; n, Number; cm, Centimeter; y, Year; kg, Kilogram.

†Mean difference of serum AMH was calculated based on preoperative and 3 months after surgery.

††Statistical tests were done based on mean difference of serum AMH pre- and 3 months after surgery.

*Paired t-test.

**One-way ANOVA test.

***Student**’**s t-test.

**Figure 2:**
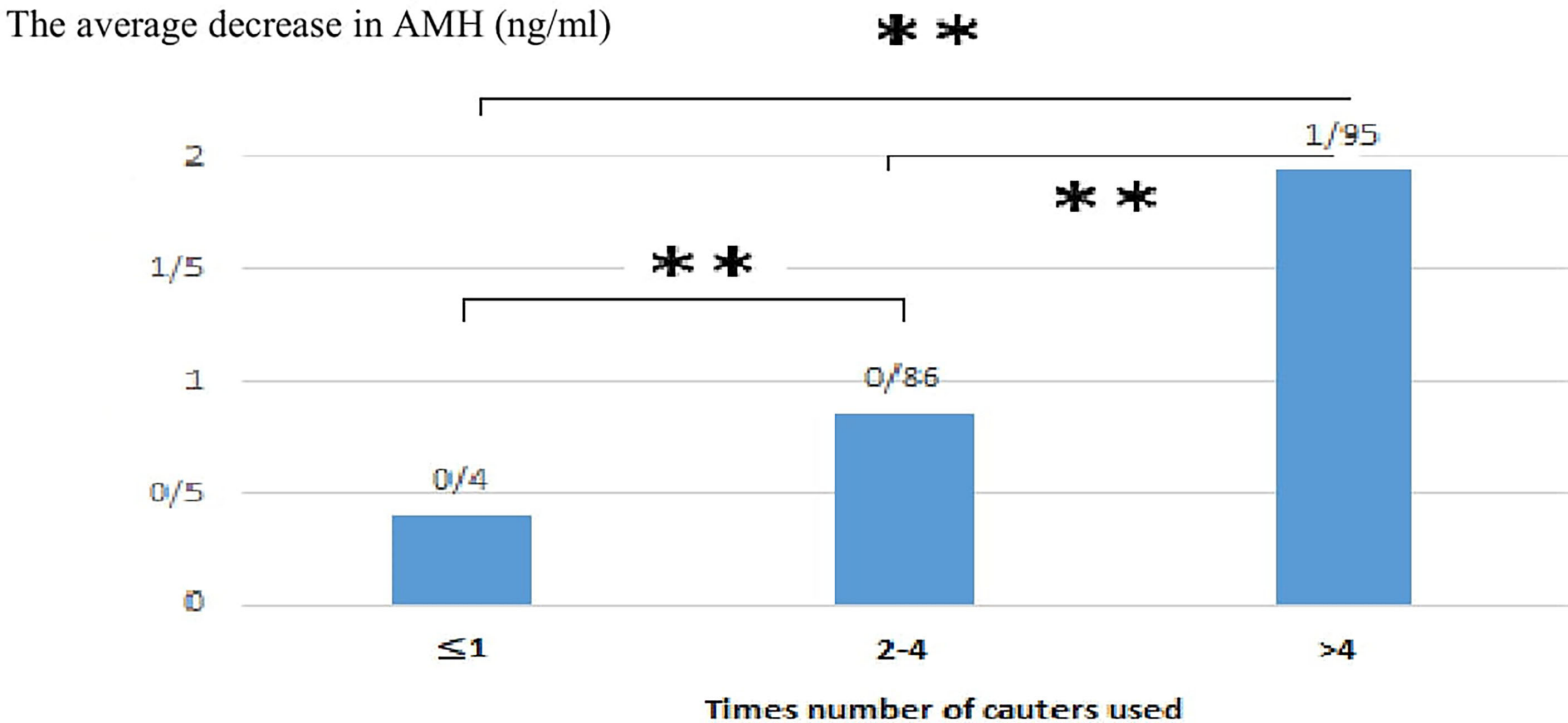
Tukey post hoc test result to compare the average decrease of AMH in different subgroups based on the number of cauterization used. p<0.001**.

As the secondary outcome of the study, serum AMH level was compared in sub-classification of the number of cauterizations, size, location, type of cysts, age, and BMI. As shown in **Table 2**, there was a negative significant relationship between the number of cauterizations used and the decrease in the serum AMH level (p ≤ 0.001). This indicates that an increase in the number of cauterizations leads to a decrease in the serum AMH levels. The lowest rate of decrease was observed in women by 1 cauterization number (0.42 ± 0.4 ng/ml), while the highest rate of decrease was in the group by > 4 cauterization number (1.95 ± 0.76 ng/ml). There was a significant difference between all study groups (p ≤ 0.001). The average decrease of serum AMH level in different subgroups based on the number of cauterizations used is shown in **Figure 2**.

The mean decrease of serum AMH level in bilateral involvement (2.08 ± 0.83 ng/ml) was significantly higher than in unilateral involvement (1.21 ± 0.91 ng/ml) (p ≤ 0.01) (**Table 2**). There was a significant difference between cyst type and a decrease in serum AMH level (p ≤ 0.001). The highest rate of decrease was observed among patients with endometriosis (2.09 ± 0.7) followed by serous cystadenoma (1.51 ± 0.65), and dermoid (0.79 ± 0.65) (**Table 2**).

There was no statistically significant relationship between cyst size, age of patients, and BMI with the rate of the decrease in serum AMH level (p = 0.34), (p = 0.61), and (p = 0.38) respectively. Comparison of the serum AMH pre and 3 months postoperative based on subgroups are summarized in **Table 2**.

In the present study, the multiple linear regressions model results showed that the number of cauterizations and bilaterality are two significant variables in predicting the amount reduction of serum AMH levels. The multiple linear regressions model result is summarized in **Table 3**.

**Table 3:** The predictors of the amount reduction of serum AMH levels by the multiple linear regressions model.

Coefficients^a^								
Model		Unstandardized Coefficients		Standardized Coefficients	t	Sig.	95.0% Confidence Interval for B	
	B	Std. Error	Beta				Lower Bound	Upper Bound
1	(Constant)	.599	.266		2.249	.026	.071	1.127
	Number of cauterization	-.217	.022	-.666	-10.000	.000	-.260	-.174
	Bilaterality	-.789	.262	-.201	-3.015	.003	-1.307	-.270

a. Dependent Variable: mean difference AMH.

*AMH, Anti-Mullerian Hormone.

## Discussion

Effect of laparoscopic cystectomy on the serum AMH levels is still debatable (21–23). Therefore, this study aimed to evaluate the effect of laparoscopic cystectomy on ovarian reserve in patients with ovarian cysts.

As the main outcome of the present study, the mean serum level of AMH had declined significantly 3 months after surgery as compared to before surgery. This result is supported by other studies (17, 18, 22, 24–31). On the other hand, this outcome was inconsistent with the results of the research of Amooee and colleagues in Iran (32). Moreover, the results of Haghgoo et al. in Iran showed that serum AMH levels increase in long-term follow-up of patients with endometrioma after laparoscopy (33). The reason for the discrepancy between the results of the present study and the results of the research of Amooee et al., and Haghgoo et al. is due to the difference in methods for homeostasis (bipolar coagulation or suture) and length of the follow-up period (32, 33). But in general, the results of the present study are in line with and previously published studies (a total of more than 30 studies with more than 1200 participants) showed a significant reduction in serum AMH level after laparoscopy of ovarian cysts. The results of studies have shown that various mechanisms can lead to a decrease in ovarian reserve due to laparoscopic surgery (34–36). This is because healthy ovarian tissue may be inadvertently removed during ovarian cystectomy due to the lack of a clear demarcation between the cyst and the ovary, leading to the destruction of follicles (23).

As the secondary outcome of the present study, there was a significant relationship between the number of cauters used, the location (unilateral or bilateral), and the type of cyst with the amount of serum AMH level drop. However, the results of our study showed that there was no statistically significant relationship between cyst size and reduction in serum AMH level, which is not in line with the results of Ali et al. (24), Wang et al. (26), Amooee et al. (32) and Alborzi et al. (20). It was probably due to the difference in how the cyst size was classified in the present study in comparison with other studies. Therefore, in the present study cysts were classified into two groups based on their size (< 7cm and ≥ 7cm). In the study of Amooee and colleagues, cysts were classified into two groups as ≥ 9 cm and < 9 cm. Alborzi et al. classified cysts into two groups as ≥ 3 cm and < 3 cm. It was claimed that with increasing cyst size, the amount of serum AMH level in the third month after surgery would be significantly reduced (20, 24, 32). On the other hand, resection of cysts with smaller sizes may be more difficult and may be associated with a higher chance of ovarian tissue damage, follicular destruction, vascular damage, and the resulting inflammatory processes (37). Therefore, it is suggested that to achieve a suitable number for segmentation based on the size of different cysts, additional research should be done in this regard and instead of two-group classification, multi-group classification should be used for the cyst size variable.

In the present study, the rate of serum AMH level reduction in patients with bilateral involvement was significantly higher than the unilateral type, which was consistent with the results of Ono et al. (17), Wang et al. (26), Satio et al. (28). Also, these results were inconsistent with the results of Alborzi et al. (20). It seems that this decrease is more in bilateral cysts due to more ovarian damage during surgery as compared to unilateral cysts. Furthermore, the results of the present study showed that by increasing the number of cauterizations used, the level of serum AMH reduced significantly. Previously, no research has directly evaluated this variable (number of cauterizations used), and this is one of the valuable points of this study. A study by Hartono et al. in Indonesia showed that there was no statistically significant difference between the reduction in serum AMH level in the group undergoing cauterization compared to the group undergoing stitching (19). On the contrary, the results of Zhang et al. in China showed that the use of cautery significantly reduces serum AMH level after surgery (38). Because cauterization uses heat energy to maintain homeostasis in tissues, this heat causes damage to the surrounding tissues in addition to stopping bleeding from the vessel. This resulting heat energy is transferred to more cells (higher heat transfer coefficient) and causes thermal damage, ovarian vascular damage, and postoperative inflammatory response which lead to an increase in the relative risk of depleting ovarian reserves. Therefore, it is suggested that additional research has to be done on this issue to avoid unnecessary cauterization as much as possible.

Additionally, the present study outcomes showed that the mean decrease in serum AMH level among participants with endometrioma was significantly higher than the “serous cystadenoma”, “dermoid cyst”, and “other” groups. Ergun et al. (22) and Hartono et al. (19) reported that endometriosis with different mechanisms such as inflammatory processes and intensification of oxidative stress mechanisms in the tissue causes a further decrease in serum AMH levels than other types of ovarian cysts.

In addition, our study results showed that no statistically significant relationship exists between age and serum AMH level decrease in participants, which is inconsistent with the results of Ono et al. (17) and Ali et al. (24). This may be due to the average age of participants in the present study is lower than other existing studies.

The main strength of the present study was the first study addressing the number of cauterizations during surgery and serum AMH level changes. Although the present study provided important data, the limitations are worthy of mention. One of the limitations of the study was its short follow-up period (i.e., 3 months). It is suggested that further research should be conducted with more follow-up in other centers.

Based on the results of this study and considering the importance of ovarian reserves for the fertility of patients, the results of this study along with other studies can be used to select qualified patients and use more appropriate techniques to reduce the amount of serum AMH level in laparoscopic surgery of ovarian cysts.

## Conclusion

The present study outcomes showed that, the serum level of AMH decreases significantly after cystectomy, and this decrease can be associated with factors such as the type of cyst, the type of involvement, and the number of cauterizations used during the operation. It seems that the appropriate choice of patients who are candidates for ovarian cyst surgery is by considering their fertility conditions before surgery, careful consultation with patients seeking future fertility, consultation with infertility specialists and even considering freezing before the above operations, using the suturing technique for establishing homeostasis instead of cauterization and reducing the number of cauterizations during surgery can lead to better functional results after the surgery.

## Data availability statement

The original contributions presented in the study are included in the article/supplementary material. Further inquiries can be directed to the corresponding author.

## Ethics statement

The studies involving human participants were reviewed and approved by Kerman University of Medical Sciences with the ethics code IR.KMU.AH.1399.40. The patients/participants provided their written informed consent to participate in this study. Written informed consent was obtained from the individual(s) for the publication of any potentially identifiable images or data included in this article.

## Author contributions

GM and MS, designed the study and gathered the data. AS, MS, HS, LA, and IA analyzed the data. All authors discussed the results and commented on the manuscript.

## Funding

The study was funded by Kerman University of Medical Sciences (Grant number 99-02-218).

## Acknowledgments

The authors thank all patients who kindly agreed to participate in the study.

## Conflict of interest

The authors declare that the research was conducted in the absence of any commercial or financial relationships that could be construed as a potential conflict of interest.

## Publisher’s note

All claims expressed in this article are solely those of the authors and do not necessarily represent those of their affiliated organizations, or those of the publisher, the editors and the reviewers. Any product that may be evaluated in this article, or claim that may be made by its manufacturer, is not guaranteed or endorsed by the publisher.
